# Localization of TrkB and p75 receptors in peritoneal and deep infiltrating endometriosis: an immunohistochemical study

**DOI:** 10.1186/s12958-016-0178-5

**Published:** 2016-08-12

**Authors:** Agung Dewanto, Jozsef Dudas, Rudolf Glueckert, Sylvia Mechsner, Anneliese Schrott-Fischer, Ludwig Wildt, Beata Seeber

**Affiliations:** 1 Department of Gynecological Endocrinology and Reproductive Medicine, Medical University of Innsbruck, Anichstrasse 35, Innsbruck, 6020 Austria; 2 Department of Obstetrics and Gynecology, Gadjah Mada University, Yogyakarta, Indonesia; 3 Department of Otorhinolaryngology, Medical University of Innsbruck, Innsbruck, Austria; 4 Endometriosis Centre Charité, Department of Gynecology - Campus Benjamin Franklin, Charité Universitätsmedizin Berlin, Berlin, Germany

**Keywords:** Neurotrophins, BDNF, Endometriosis, Immunohistochemistry

## Abstract

**Background:**

The roles of the neurotrophins NGF (Neurotrophic growth factor) and BDNF (brain-derived neurotrophic factor) in neuronal growth and development are already known. Meanwhile, the neurotrophin receptors TrkA (tropomyosin related kinase A), TrkB, and p75 are important for determining the fate of cells. In endometriosis, this complex system has not been fully elucidated yet. The aim of this study was to evaluate the expression and location of these neurotrophins and their receptors in peritoneal (PE) and deep infiltrating endometriotic (DIE) tissues and to measure and compare the density of nerve fibers in the disease subtypes.

**Methods:**

PE lesions (*n* = 20) and DIE lesions (*n* = 22) were immunostained and analyzed on serial slides with anti-BDNF, −NGF, −TrkA, −TrkB, −p75,-protein gene product 9.5 (PGP9.5, intact nerve fibers) and -tyrosine hydroxylase (TH, sympathetic nerve fibers) antibodies.

**Result:**

There was an equally high percentage (greater than 75 %) of BDNF-positive immunostaining cells in both PE and DIE. TrkB (major BDNF receptor) and p75 showed a higher percentage of immunostaining cells in DIE compared to in PE in stroma only (*p* < 0.014, *p* < 0.027, respectively). Both gland and stroma of DIE lesions had a lower percentage of NGF-positive immunostaining cells compared to those in PE lesions (*p* < 0.01 and *p* < 0.01, respectively), but there was no significant reduction in immunostaining of TrkA in DIE lesions. There was no difference in the mean density of nerve fibers stained with PGP9.5 between PE (26.27 ± 17.32) and DIE (28.19 ± 33.15, *p* = 0.8). When we performed sub-group analysis, the density of nerves was significantly higher in the bowel DIE (mean 57.33 ± 43.9) than in PE (mean 26.27 ± 17.32, *p* < 0.01) and non-bowel DIE (mean 14.6. ± 8.6 *p* < 0.002).

**Conclusions:**

While the neurotrophin BDNF is equally present in PE and DIE, its receptors TrkB and p75 are more highly expressed in DIE and may have a potential role in the pathophysiology of DIE, especially in promotion of cell growth. BDNF has a stronger binding affinity than NGF to the p75 receptor, likely inducing sympathetic nerve axonal pruning in DIE, resulting in the lower nerve fiber density seen.

**Electronic supplementary material:**

The online version of this article (doi:10.1186/s12958-016-0178-5) contains supplementary material, which is available to authorized users.

## Background

Endometriosis is diagnosed by the presence of endometrial glands and stroma outside of the uterine cavity. These endometriotic lesions are predominantly found in the pelvis, namely on the peritoneum, ovaries, myometrium of the uterus (adenomyosis) and the bowel. The gold standard for the diagnosis of endometriosis is surgical visualization, which is commonly accomplished with laparoscopy, followed by histological confirmation of operatively excised lesions [1]. Endometriosis can be divided into two subtypes: superficial peritoneal endometriosis (PE) and deep infiltrating (DIE) disease, the latter defined by lesions located at least 5 mm beneath the peritoneal surface. The pathophysiology of DIE, including its development, innervation, and association with inflammation and vascularization, has been the subject of numerous studies. Most, but not all, conclude that these aspects differ between DIE and PE endometriosis [2–4].

In addition, multiple investigators have aimed at elucidating the mechanisms of pain generation in endometriosis to improve the understanding of endometriosis-associated pelvic pain [5, 6]. Previous studies have evaluated for the presence of nerve fibers in endometriotic lesions, comparing the innervation density between PE, DIE and normal peritoneum using specific stains for all intact-nerve fibers (Protein Gene Product 9.5,PGP9.5), sensory nerve fibers-(Substance-P), and sympathetic-nerve fibers (tyrosine hydroxylase) [7, 8]. The studies have found that: (1) there is little difference in overall nerve fiber density between PE and healthy peritoneum and (2) PE lesions have a higher density of sensory nerve fibers and a lower density of sympathetic nerves than healthy peritoneum [7]. In DIE, the density of nerve fibers stained with PGP9.5 was the highest in endometrial lesions taken from rectum compared to the density seen in peritoneal lesions and in DIE lesions taken from other locations such as the uterosacral ligament or the retro-uterine cul de sac. Surprisingly, the nerve fiber density in non-bowel DIE (cul-de-sac and uterosacral ligament lesions) was not different than that observed in peritoneal endometriosis [8].

The presence of neurotrophins, a family of proteins critical to supporting the growth and differentiation of developing neurons and to maintaining neuronal survival, has been studied in endometriosis [9]. The key components of the neurotrophin system in humans are nerve growth factor (NGF), brain-derived neurotrophic factor (BDNF), and the neurotrophins-3 (NT-3), −4/5 (NT-4/5) [10] as well as their respective receptors, the tropomyosin kinase receptors (TrkA, TrkB, TrkC) and the neurotrophin receptor p75, a member of the tumor necrosis factor (TNF) receptor superfamily. Specifically, the Trk receptors consist of: TrkA, the high affinity receptor for NGF and TrkB, the main receptor of BDNF and NT4/5. The binding of NGF and BDNF to the P75 receptor induces survival of the cell while the binding of the premature type of neurotrophin, pro-NGF and pro-BDNF, to the p75 receptor induces apoptosis in the cells [10, 11].

In endometriosis, the expression of NGF was confirmed using immunohistochemistry in the gland, stroma, [12–14] and in nerve fibers of endometriosis lesions [4, 8]. In addition, the concentration of the neurotrophin NGF has been reported by several groups to correlate with the density of nerve fibers, both in PE [4, 13, 14] as well as in DIE, including bowel endometriosis [8, 12, 15]. Furthermore, Anaf et al. reported that NGF expression was higher in the glands and stroma of adenomyosis lesions compared to PE [13], but interestingly did not differ from endometrium of disease-free controls [16].

The spatial relationship between the main neurotrophins (NGF and BDNF) and their receptors (TrkA, TrkB, p75) in histological sections from endometriosis tissue has not been extensively studied so far. Tarjanne et al. recently reported that NGF and its receptor TrkA were strongly expressed in rectovaginal endometriosis but they did not make comparisons to PE or DIE from other locations [12, 14]. No studies have evaluated the presence and location of BDNF in endometriosis lesions, although BDNF is present in eutopic endometrium from women with endometriosis.

The main objective of this immunohistochemistry study was to assess for the presence and localization of the neurotrophins NGF and BDNF and their receptors, TrkA and, TrkB and p75, in endometriosis lesions and to compare these between PE and DIE by using quantitative methods. Secondarily, we compared the density of nerve fibers in PE versus non-bowel DIE and bowel DIE.

## Methods

### Subjects

Archived endometriotic tissue samples were collected from 44 patients who had undergone surgery for pain, infertility suspected uterus malformation or other gynecologic indication. Disease was confirmed histologically by the presence of both endometrial glands and stroma in an ectopic location. We analyzed PE lesions from 20 women and DIE lesions from 22 women, 7 of whom had deep endometriosis lesions located on the bowel (*n* = 7), the remaining in non-bowel pelvic locations (*n* = 15). Endometriosis was staged surgically according to the American Society for Reproductive Medicine (ASRM) revised guidelines. The age of patients ranged from 27 to 45 years (mean age ± Standard Deviation (SD): 33.15 ± 5.79 in PE group) and 24 to 49 years (mean age ± SD: 33.54 ± 6.72 in DIE group). Medical records were reviewed to collect relevant clinical information. All subjects were confirmed to be premenopausal with regular menstrual cycles. For the women in the PE group, 8 were in the menstrual or proliferative phase and 9 in the secretory phase of the cycle, and for 3 women this information was not available. For the most of the women in the DIE, menstrual phase information was not available, while 5 were confirmed to be in the menstrual or proliferative phase. Table 1 summarizes the relevant clinical data of subjects.

**Table 1:** Clinical characteristics of subjects

Patient Information	PE	DIE
*n*	20	22
Age (mean ± SD)	33.15 ± 5.79	33.54 ± 6.72
rASRM		
I	7	0
II	10	6
III	3	11
IV	0	5
Menstrual Phase		
Proliferative/menstruation	8	5
Secretory	9	–
Unknown/missing	3	17
Pain Type/location		
Lower abdominal pain	4	7
Dysmenorrhea only	8	8
Mixed Pain	1	2
No pain	7	5
Location of DIE lesion		
Bowel (rectum, appendix, colon/sigmoid)	–	7
Non-bowel:	–	15
Paraureteral	–	2
Bladder	–	2
Pararectal/rectovagina	–	10
vagina	–	1

None of the subjects received medical therapy for endometriosis nor hormonal contraception for at least 3 months prior to laparotomy or laparoscopy for the excision of endometriosis. This study was approved by the Ethics Committee of the Medical University of Innsbruck (No. UN5130, July 2nd, 2013) and all patients gave their informed consent for research participation.

### Histological specimens

All the specimens were immediately fixed in 4 % paraformaldehyde for 12 h, and processed and embedded in paraffin according to standard protocols. Each section was cut at 3 μm thickness for 15 serial sections. Serial sections enabled us to study corresponding spatial/anatomical sites and evaluate the location of neurotrophins as well as their receptors. Routine hematoxylin and eosin (H&E) staining was performed for tissue overviews.

Immunohistochemistry of sections with antibodies directed against NGF, BDNF, TrkA, TrkB, p75, PGP9.5, and TH were performed. Immunohistochemistry was performed with an automated staining system Ventana® Roche® Discovery that ensured precise and equal treatment of each slide. This was a prerequisite for quantification of immunostaining intensities. Sections were immunohistochemically stained with the polyclonal rabbit anti-NGF (dilution: 1:400, sc-548, Santa Cruz Biotechnology, California), polyclonal rabbit anti-BDNF (dilution: 1:1200, ab101752, Abcam, England), polyclonal goat anti-TrkA (dilution: 1:240, sc-20,537 Santa Cruz biotechnology, California) polyclonal rabbit anti-TrkB (dilution: 1:60, sc-8316, Santa Cruz Biotechnology, California), polyclonal rabbit anti p75 (dilution: 1:3200, a gift from Prof. Reichardt, University California San Francisco), polyclonal rabbit anti PGP.9.5 (dilution: 1:2400, ab10404, Abcam, England) monoclonal mouse anti-tyrosine hydroxylase (dilution 1:2000, T1299, Sigma, USA). Healthy human colon and healthy human endometrium were used as positive controls for neurotrophins and receptors. Healthy human skin and healthy human colon were used as positive controls for nerve fibers. Negative controls were treated identically except that the primary antibody was replaced with IgG rabbit or goat isotype for polyclonal antibodies and IgG1 mouse for monoclonal antibody.

### Cell counting

For each study participant, one representative gland and area of stroma were identified. Once the immunohistochemistry analysis for that gland and stroma was carried out with a single antibody, it was then carried out on the same gland and stroma in the adjacent section for another antibody. With this method, we could determine the precise localization of the neurotrophins and receptors on the same gland and stroma.

Stained tissue sections were analyzed at × 20 magnification using a Tissuefax Plus system based on a Zeiss® AxioImagerZ2 Microscope (Jena, Germany). Images were acquired with the TissueFaxs (Tissue-Gnostics®, Vienna, Austria) software. The percentage of NGF-, BDNF-, TrkA-, TrkB-, and p75 –positive immunostaining cells in each endometriosis tissue specimen was quantified using HistoQuest® (Tissue-Gnostics) software. This software has been used by previous researchers [17–19] and has the advantage of being more objective than the subjective assessment by an investigator.

Histoquest® is an analytical tool used to quantify immunostaining based on single cells using the cell specific nucleus structure as the primary identification marker (hematoxylin), followed by an automatic segmentation of the immunostaining confined to the corresponding nucleus. A ring mask around this nucleus is interactively defined and set as parameter for all sections stained with a certain marker-specific channel named single reference shade. The brown staining caused by chromogen (3,3’-diaminobenzidine, DAB) is automatically separated from the blue hematoxylin staining into their optical density counterparts. The mean optical density per cell is quantified by the segmentation method.

Regions of interest (ROIs) were defined separately for glandular epithelial tissue and stromal tissue. Identification of cell types was accomplished through morphometric parameters such as the nuclear size, shape and staining intensity. A background threshold for hematoxylin staining was determined interactively. Immunostaining cut-offs were determined as well (this tool differentiates between positive and negative cells; these were set in the dot blots). All images were acquired with the same setting parameters. The representative brown color (DAB chromogen) was picked by the color picker tool. Positive staining cells were shown in the scatter gram of forward gating tool. The raw data of the analysis were imported into SPSS 21.0 (IBM, Armonk, NY, USA) for further statistical analysis. The number of NGF-, BDNF-, TrkA-, TrkB-, and p75 –positive immunostaining cells was divided by the total number of cells in each gland or stroma of endometriosis tissue (hematoxylin counterstain), yielding a percentage of staining. More detailed methodology and setting parameters of Histoquest® can be found in the Supplemental Section (Additional files 1, 2, 3, 4 and 5: Figs. S1, S2, S3, S4 and S5).

### Nerve fiber density

Each stained endometriosis section was imaged at × 40 magnification using the above described TissueFaxs Plus systems. The evaluation area was randomly set to 1 mm^2^ surrounding the nerve fiber located nearest to the lesion. The area of interest was manually highlighted with a tool provided by HistoQuest® software to comprise an area of 1 mm^2^ as shown in Additional file 6: Fig. S6. Thus, the total nerve fiber density was calculated by averaging the amount of nerve fibers defined by PGP9.5 positive staining in 3 different areas of endometriosis of 1 mm^2^ each (Additional file 6: Fig. S6). The same method was used to calculate the density TH-positive staining sympathetic nerve fibers.

### Statistical analysis

The Mann-Whitney *U* test was used to evaluate the difference between the percentage of positive immunostaining cells in PE and DIE. The student’s *t*-test was used to compare mean nerve fiber density between groups. Pearson correlation was used to analyze the correlation between neurotrophins and the nerve fibers. *P* < 0.05 was considered statistically significant at 95 % of confidence interval.

## Results

### Staining for BDNF and TrkB

**Fig. 1:**
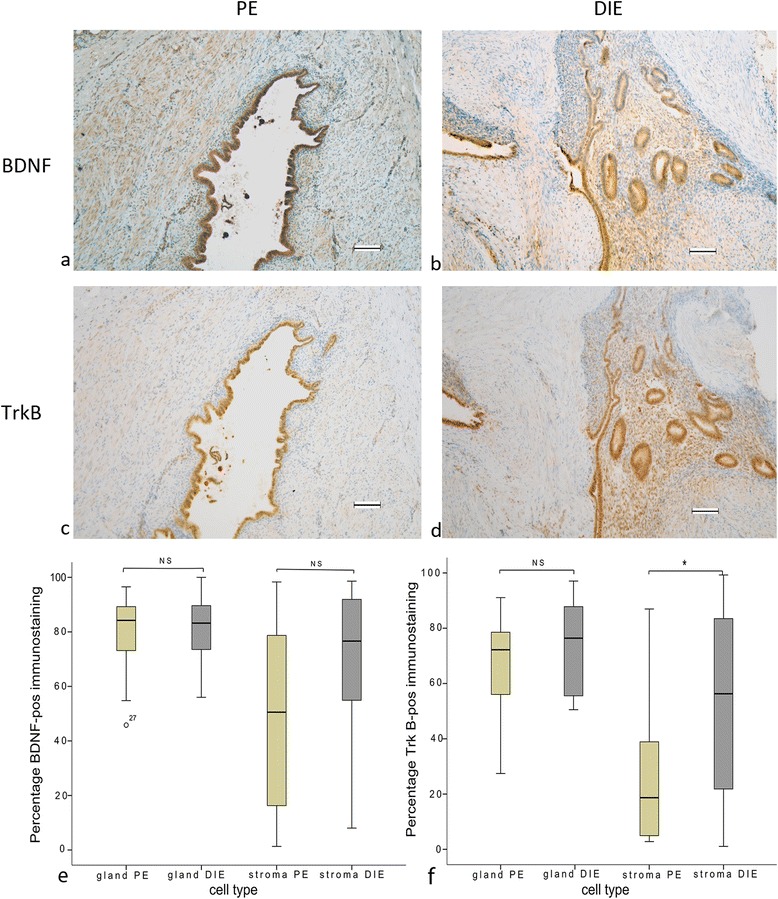
Formalin-fixed paraffin-embedded serial section of PE (**a**, **c**) and DIE (**b**, **d**) stained for BDNF (**a**, **b**) and TrkB (**c**, **d**). Original magnification × 100. The percentage TrkB positive immune staining cells in stroma showed a significant difference between stroma groups* *P* < 0.014 (**f**), but not significant difference in other groups (*P* > 0.05) (**e**). Scale bar, 100 μm

BDNF exhibited strong staining intensity in both gland and stroma cells of PE and DIE, with no differences seen (Fig. 1e). Nonetheless, in stroma, there was a significantly higher percentage of cells expressing TrkB positive immunostaining in DIE compared to PE. No such difference in TrkB staining was seen in glands of PE and DIE (Fig. 1f).

### Staining for NGF and TrkA

**Fig. 2:**
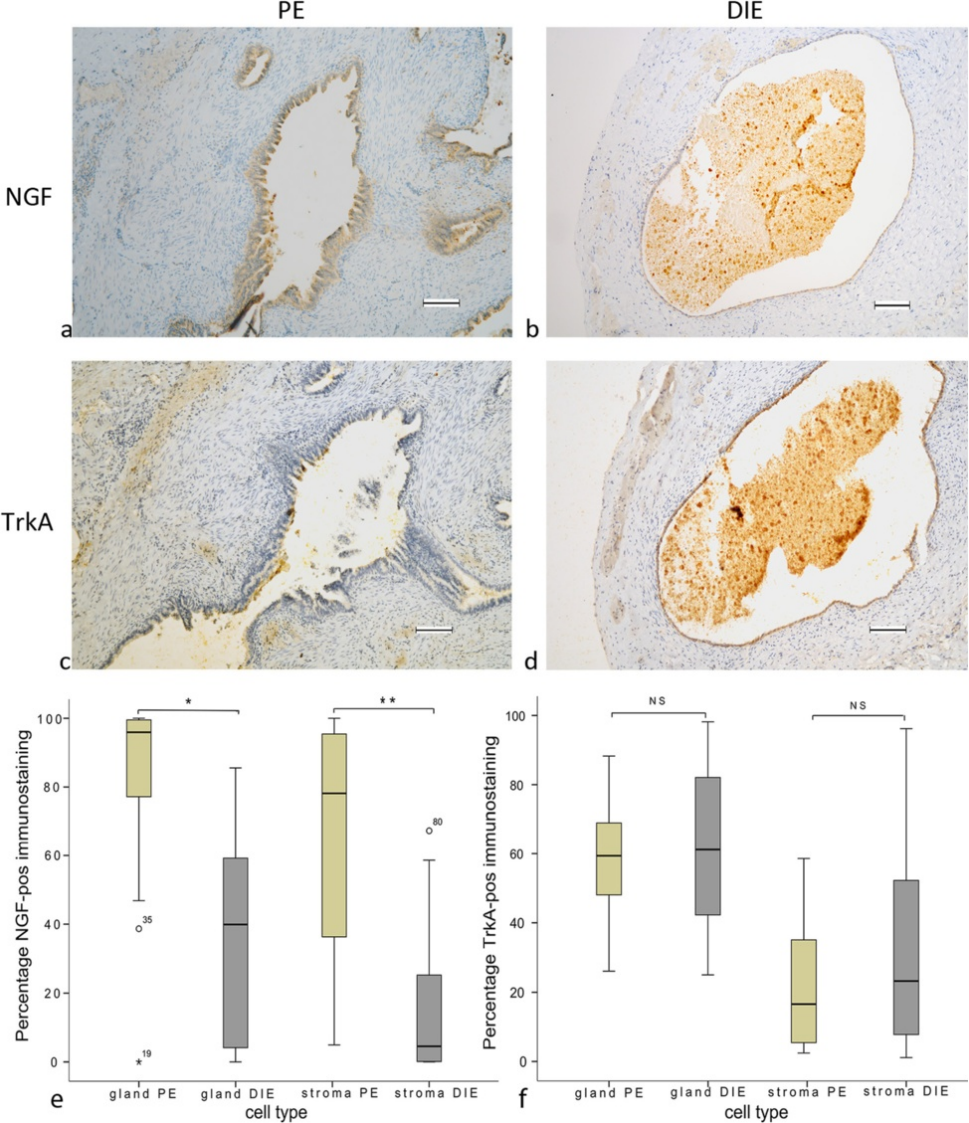
Formalin-fixed paraffin-embedded serial section of PE (**a**, **c**) and DIE (**b**, **d**) stained against NGF (**a**, **b**) and TrkA (**c**, **d**). Original magnification × 100. The percentage NGF positive immune staining cells in stromal cells showed significant difference (*P < 0.00, **P < 0.00) in glandular epithelial cells and stromal cells between PE and DIE (**e**). No differences were seen for TrkA-positive immunostaining (**f**). High degree of positive staining was observed in the lumen of the endometriotic gland reflecting immune cells (**b**, **d**). Scale bar, 100 μm

The expression of NGF positive immunostaining cells was higher in both the gland and stroma of PE compared to the gland and stroma of DIE, as shown in Fig. 2. Nevertheless, staining for TrkA, the main receptor of NGF, showed no differences in glands or stroma between PE and DIE (Fig. 2f).

### Staining for p75

**Fig. 3:**
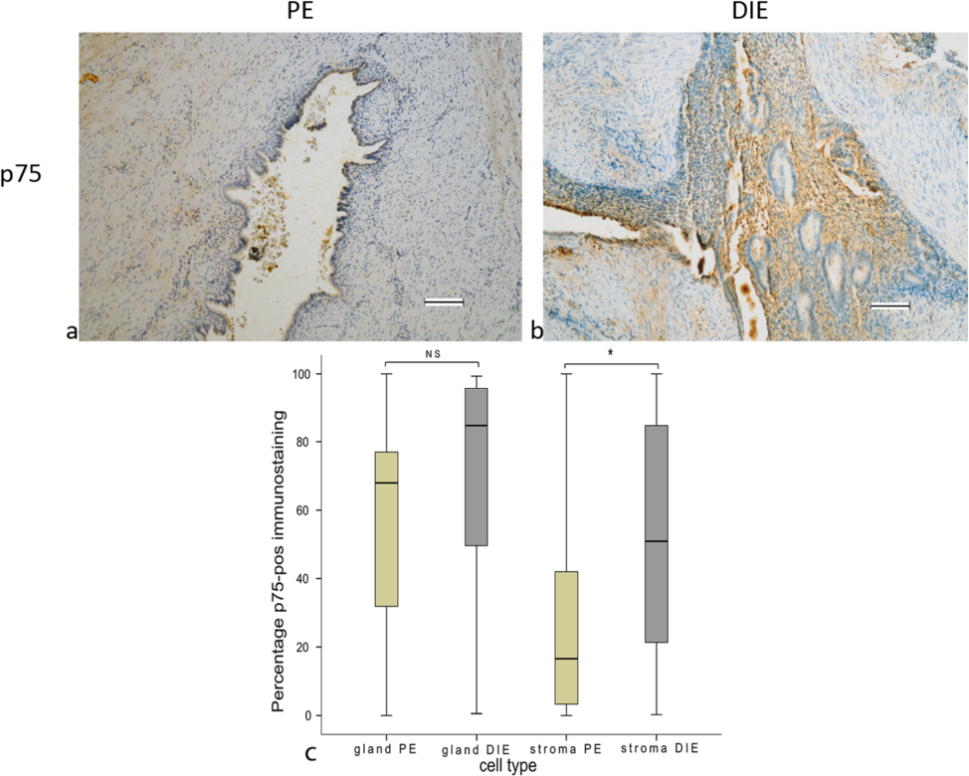
Formalin-fixed paraffin-embedded serial section of PE (**a**) and DIE (**b**) stained for p75 (**a**, **b**). Original magnification × 100. The percentage p75 positive immune staining cells in stromal cells showed a significant difference * *p* < 0.027 between PE and DIE (**c**). Scale bar, 100 μm

There was a high percentage of p75-positive immunostaining seen in the endometrial glands of both PE and DIE. In the stroma, there was a significantly higher percentage of p75 staining in DIE compared to PE (Fig. 3c).

### Staining nerve fibers for PGP9.5 in PE, bowel DIE and non-bowel DIE

**Fig. 4:**
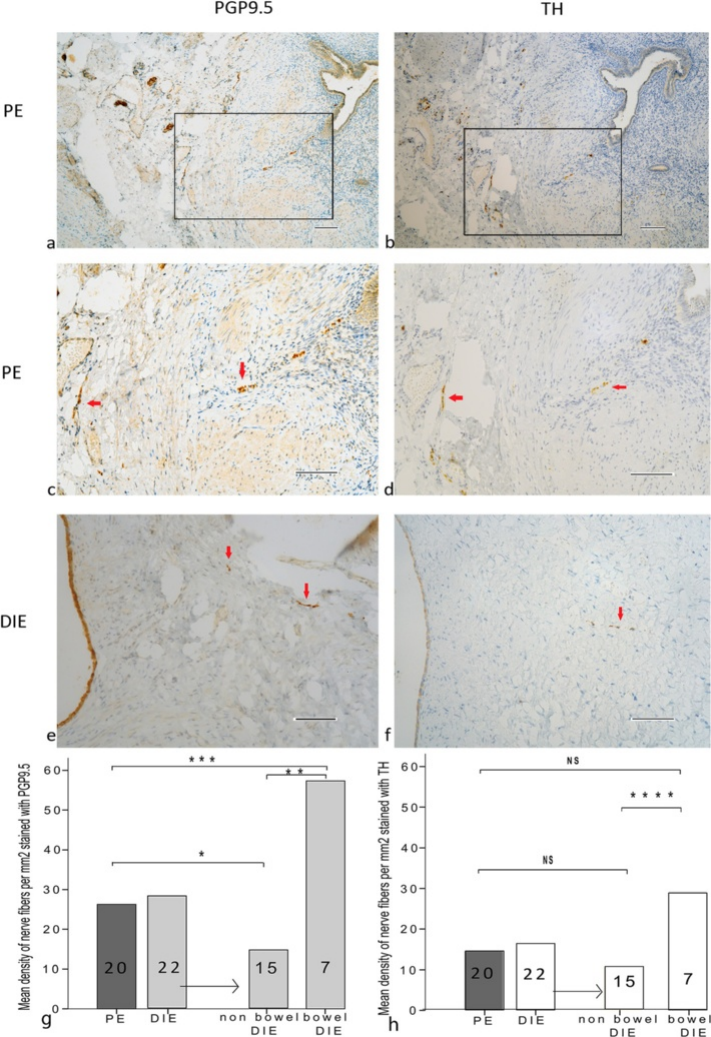
Formalin-fixed paraffin-embedded serial section of PE (**a**, **b**, **c**, **d**) and DIE non bowel (**e**, **f**) stained for PGP9.5 (**a**, **c**, **e**) and . Original magnification × 100 (**a**, **b**) and × 200 (**c**, **d**, **e**, **f**). Picture c and d are an inset from (**a**) to (**b**) respectively. Red arrow showed nerve fibers. Nerve fibers stained with PGP9.5 were significantly different between PE and non-bowel DIE endometriosis *p < 0.041, between PE and bowel **p < 0.01 and between non-bowel and bowel *** *p* < 0.002 **g**. Nerve fibers stained with TH were not significantly different between PE and non-bowel DIE endometriosis *P* = 0.21, between PE and bowel *P* = 0.06 and significantly different between non-bowel and bowel ****p < 0.04 (**h**). Scale bar, 100 μm

Nerve density stained with PGP9.5 was similar between PE (26.27 ± 17.32) and DIE (28.19 ± 33.15), *p* = 0.81. When we performed sub-group analysis and separately evaluated the bowel and non-bowel DIE, the density of nerves of was significantly higher in the bowel DIE (57.33 ± 43.9) than in the PE (*p* < 0.01) and non-bowel DIE (14.6. ± 8.6 *p* < 0.002) (Fig. 4g).

### Staining nerve fibers using TH compared to PGP9.5 in non-bowel DIE

Mean density of sympathetic nerve fibers stained with TH was not different between PE and DIE. When the DIE group was split to bowel and non-bowel endometriosis, the pattern of mean density of nerves for each group stained with TH was similar to that seen with PGP9.5, with markedly higher concentration of sympathetic nerves in bowel DIE, and similarly lower sympathetic innervation of non-bowel DIE and PE (Fig. 4h).

### Staining for nerve fibers using PGP9.5 in DIE of bowel (colon)

**Fig. 5:**
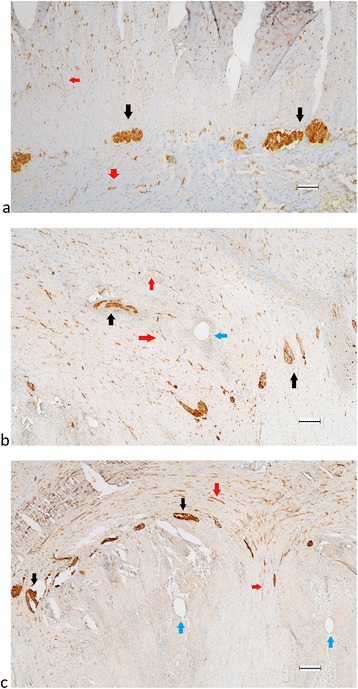
Ganglion in plexus myentericus of healthy human colon (**a**) and colon endometriosis lesion (**b**, **c**) stained with PGP9.5. Original magnification 40 ×. Black arrows showed ganglions, red arrows showed nerve fibers, blue arrows showed endometriosis. Irregular form of plexus myentericus depicted in picture **b** and **c**. Scale bar, 200 μm

The plexus myentericus is located in between two muscle layers, circular and longitudinal fibers. In normal colon (Fig. 5a) the plexus was seen as a regular straight line interrupted by ganglia. In colon infiltrated by endometriosis, the plexus myentericus lost its longitudinal configuration, as depicted in Fig. 5b and c. Furthermore, the regularity for the muscle layers was lost and their structure became aberrant.

### The correlation between neurotrophin expression and nerve fibers density

We performed correlation analyses between NGF and BDNF immunostaining and nerve fiber density (PGP9.5 staining) for both gland and stroma, and for the subtypes of endometriosis (PE and non-bowel DIE). We chose to exclude bowel DIE in these analyses because of the inability to differentiate between the intrinsic and extrinsic innervation, thus making endometriosis-specific nerve growth impossible to study.

When both subtypes of endometriosis tissues were taken together (PE and DIE), there was no significant correlation between BDNF-positive staining and nerve density assessed by PGP9.5, neither for gland nor stroma (data not shown). When only the gland was assessed, there was a negative correlation seen only in non-bowel DIE (*P* < 0.007, *r* = −0.618). We then did analogous correlations for NGF and PGP9.5, BDNF and TH and also NGF and TH.

There were no significant correlations between NGF-positive staining and nerve fiber density assessed by PGP9.5 as can be seen in Table 2 (NGF vs PGP9.5) and nerve fiber density stained with TH, except in gland PE that showed a significant negative correlation (*P* = 0.038, *r* = −0.406). We found a negative correlation between BDNF-positive staining and the density of nerve fibers stained with TH in the gland of non-bowel DIE (*P* < 0.036, *r* = −0.478) (Table 2).

**Table 2:** The correlation between BDNF-positive immunostaining and the density of nerve fibers stained with PGP9.5

	Gland		Stroma	
BDNF vs PGP9.5	*P* value	r	*P* value	r
PE	0.196	−0.202	0.324	0.109
DIE non-bowel	**0.007**	**−0.618**	0.426	−0.053
NGF vs PGP9.5				
PE	0.218	−0.185	0.248	0.149
DIE non-bowel	0.187	0.247	0.404	−0.069
BDNF vs TH				
PE	0.160	−0.235	0.299	0.125
DIE non-bowel	**0.036**	**−0.478**	0.368	−0.095
NGF vs TH				
PE	**0.038**	**−0.406**	0.483	0.010
DIE non-bowel	0.322	0.130	0.289	−0.156

Statistically significant differences are shown in bold

### Staining for neurons (PGP9.5), p75 and trks receptors in endometriosis in submucosa of colon

The submucosal plexus in the bowel strongly stained with p75 and TrkB antibodies, and faintly stained with TrkA antibody. The submucosal plexus has an important role in innervating mucous glands of the bowel. As depicted in Additional file 7: Fig. S7, endometrial glands were surrounded by small nerve fibers.

**Fig. 6:**
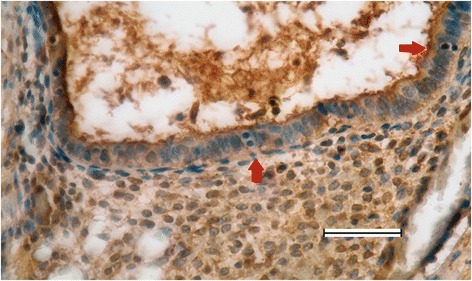
Mitotic activity in glandular epithelial cells surrounded by p75 immunostaining cells. Original magnification was × 400. Scale bar, 100 μm

In glandular epithelial cells surrounded by p75 immunostaining cells typical formations of mitosis telophase with two separated cell nuclei were identified (Fig. 6).

## Discussion

### Cell growth and development

Based on the high degree of staining for BDNF in both glands and stroma of PE and DIE, this neurotrophin appears to play a role in the innervation of endometriosis. TrkB, the main receptor of BDNF is expressed more highly in glands than in stroma, and highest in the stroma of DIE, but with high variability. The other receptor for BDNF, p75, showed similar expression, namely high expression in glands of PE and DIE, moderate expression in stroma of DIE and relatively low expression in stroma of PE. BDNF mRNA expression has previously been shown in eutopic endometrium of women with endometriosis [20] and also in normal human and mammalian uterus as well as endometrium (glandular epithelium and stroma) [21]. We confirm, for the first time in histological sections, that BDNF is present in endometriosis lesions, both DIE and in peritoneal lesions.

NGF and BDNF are both important for axonal growth of sensory neurons, but each induces growth of a different type of sensory neuron. In vitro studies in chick dorsal root ganglion showed that neuronal growth cones turned and migrated under NGF-coated beads through the expression of TrkA receptors [22]. Another study showed that visceral afferent neurons in the nodose/petrosal sensory ganglion complex innervated vascular afferents that express high levels of BDNF in the development of arterial baroreceptors. The survival of these neurons was reduced by TrkB-Fc blocking [23]. Analogous mechanisms may be responsible for inducing sensory neuron growth in endometriosis lesions via both TrkA and TrkB.

BDNF and NGF play different roles in sensory nerve development, with BDNF influencing axonal branching and the growth of lathellipodia and NGF influencing axonal elongation of sensory neurons from the dorsal root ganglion [24]. Furthermore, endometriosis is an estrogen-dependent disease in which lesions stimulate their own growth by producing estrogen via aromatase activity [25] and also via the mechanism of tissue injury and repair [26]. Wessels et al. showed that estrogen exposure may activate BDNF-TrkB pathways in a mouse model, exerting wide ranging effects such as neural development, cell differentiation, growth and maintenance, angiogenesis, proliferation, and resistance to apoptosis [17]. In clinical studies in humans, Wessels et al. reported that that plasma BDNF concentrations were significantly higher in women with endometriosis than in controls, whereas other neurotrophins, NGF and NT4/5, were not different [18].

It is known that estrogen may have local proliferative actions as well as neuromodulatory effects on the innervation of endometeriosis [19]. In this retrospective study, we were not able to fully account for the menstrual cycle phase at which the histological sample was obtained. However, in a recent report, there was no effect of menstrual cycle phase on circulating BDNF levels in women with endometriosis [18]. In mice, estrogen exposure after ovariectomy significantly increases uterine BDNF, but the hormonal fluctuations of the murine estrous cycle do not [17]. Importantly, none of the women in this study used hormonal medication for at least 3 months prior to sample collection, as hormonal treatment has been shown to decrease the nerve fiber density in peritoneal endometriosis lesions [27].

We found that NGF was higher in PE than in DIE, in both glands and stroma. Previous studies looked only at endometrioma, adenomyosis, peritoneal lesions [13, 28, 29] and eutopic endometrium from endometriosis patients [20]. The main receptor of NGF, TrkA was not different between PE and DIE in both locations. These results differ from those of Anaf et al. who found that deep adenomyotic lesions had higher expression of NGF immunohistochemically than peritoneal endometriosis [13] and imply that DIE and adenomyosis are not comparable entities when it comes to neurotrophin expression.

We found a low NGF expression as well as low nerve fiber density in DIE, especially in non-bowel DIE. Perhaps, the loss of NGF-TrkA signaling transduction causes failure of NGF-dependent neuron to survive [30]. Furthermore, in sensory developmental studies, NGF is important for nociceptor development and BDNF is important for mechanoreceptor development [31]. Our results are consistent with those of Arellano et al. who implicated the peritoneum as an important location for pathogenesis and pain generation in endometriosis [32]. Thus, our results support the concept that NGF is involved in neuronal development and likely pain generation from peritoneum in endometriosis [12, 13, 32].

Many studies have evaluated the role of Trk receptors in the invasiveness or degree of progressiveness in cancer. TrkA receptors appear to promote the growth and metastasis in breast cancer [33, 34], while TrkB receptors promote invasion in choriocarcinoma cells [35]. We found that TrkB expression was high in DIE, especially in stromal cells. This pattern is similar to that seen for p75 expression, but not for TrkA expression. This finding could be secondary to an effect of estrogen [17]. Therefore, we speculate that p75 receptor is involved in survival rather than apoptosis in endometriosis.

### Bowel innervation

On first analysis, it appears that there is no difference in mean nerve density between PE and DIE. However, critical differences appear when DIE from bowel and non-bowel locations was evaluated separately, as also shown by Wang [8]. We found that the sub-group of non-bowel DIE endometriosis actually had a lower density of innervation than the PE.

The DIE from bowel showed markedly higher innervation, both total nerve density as well as the density of sympathetic nerves stained with TH. The innervation of bowel is highly complex, stemming from both intrinsic (from the enteric nervous system) as well as extrinsic nerves (from autonomic nervous system—parasympathetic and sympathetic nervous system). In addition, the intestine has sensory afferents originating from the vagus nerve (nodose ganglion) [36, 37] and sensory afferents originating from dorsal root ganglion [38]. A previous study likewise demonstrated that normal bowel has a rich innervation base on nerve fiber density area stained with PGP9.5 and other markers [39, 40]. The plexus of Auerbach and Meissner (submucosal plexus) likewise express PGP9.5, and may be damaged by the invasion of an endometriosis lesion, as we also showed [41–43]. Thus, it is very difficult and likely error-prone to compare the endometriosis-influenced innervation of PE to bowel endometriosis because the intrinsic and extrinsic innervations of bowel cannot be differentiated with immunohistochemistry. In future studies, investigators should focus on DIE from non-bowel sources when making comparisons to PE.

Table 3 summarizes the relative abundance of NGF, BDNF, TrkA, TrkB, p75, and nerve fibers densities for PE, non-bowel and bowel DIE.

**Table 3:**
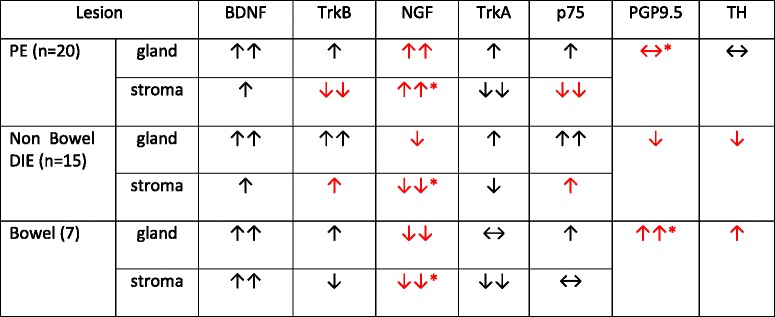
Relative abundance of the neurotophins NGF and BDNF, their receptors and nerve fibers in PE and DIE from non-bowel sources

Red arrows and * highlight statistically significant differences for each neurotrophin/receptor/nerve fiber in gland or stroma

### Correlation between cell expressing neurotrophins and the nerve fibers density

BDNF showed a negative correlation to total nerve fibers stained with PGP9.5 in glands of DIE non-bowel only, but not in other sites and not in the stroma. These results may reflect the phenomenon of axonal pruning, through which BDNF may induce pruning in neurons and thus lead to a lower nerve fiber density in the tissue. Our results are consistent with those of Singh et al. who showed that BDNF via p75 receptor influenced the development of sympathetic axon pruning, despite the simultaneous presence of NGF at the same site [44].

We found no correlation between NGF and nerve fiber density, as also reported by others [45, 46]. NGF competes for the same axons as BDNF to determine the fate of nerve fibers/neurons. In in vitro studies, the pruning/apoptosis effects of BDNF override the growth effects of NGF. Induction of pruning is stronger than the induction of growth [44]. This would explain why NGF-positive immunostaining did not correlate to a higher density of nerve fibers but instead there was a negative correlation between BDNF and nerve fiber density seen in DIE.

We did find a negative correlation between BDNF-positive staining and the density of nerve fibers stained with TH in the gland of non-bowel DIE. These results are consistent with the study by Krizsan-Agbas et al. which showed in a rat model that BDNF suppresses sympathetic neurite growth and that this effect is mediated by estrogen [45].

### Semi-automated counting

To our knowledge, ours is the first study to employ a semi-quantitative method to analyzing immunohistochemical staining in endometriosis [47]. While the software used does not replace the skills and expertise of the experienced human observer (pathologist), it is gaining popularity in the research and clinical setting [48]. The emergence of computerized image analysis systems for accurate analysis of immunohistochemistry specimen is increasingly needed. In breast cancer, a disease where the degree of estrogen receptor and progesterone receptor expression predicts outcome, Walker et al. argue that computerized image analysis systems present more accurate means of quantification. It is well accepted that manual counting is time consuming [47] and automated imaging methods are immune to fatigue and subjectivity [49].

## Conclusions

The neurotrophins and their receptors are part of a complex signaling system that are present in endometriosis. We showed that endometriotic lesions, especially epithelial glandular cells and stromal cells, express neurotrophins BDNF and NGF and their receptors, TrkA, TrkB and p75. The spatial arrangement of these agonists and receptors suggests an autocrine function in endometriosis, though a clear causative picture does not emerge given the redundancy and complexity of the signaling system. BDNF has a stronger binding affinity than NGF to the p75 receptor, likely inducing sympathetic nerve axonal pruning in DIE, resulting in the lower nerve fiber density seen in DIE. The differences in neurotrophin expression between PE and DIE may reflect the differencing innervations and cell fates, namely growth and infiltration and have been shown to be hormone-mediated. Thus, in future studies, differences in local estrogen action as measured by the distribution of estrogen receptors in co-localization with the BDNF neurotrophin signaling system and the density of nerves should be evaluated to more closely delineate this complex system and to further explain differences between PE and DIE.

## Abbreviations

ASRM: American Society of Reproductive Medicine; BDNF: brain-derived neurotrophic factor; DAB: 3,3’-diaminobenzidine; DIE: deep infiltrating endometriosis; H&E: hematoxyline and eosin; NGF: nerve growth factor; NT-3: neurotrophins-3; NT-4: neurotrophins-4; NT-5: neurotrophins-5; PE: Peritoneal endometriosis; PGP9.5: Protein Gene Product 9.5; ROI: region of interest; TH: tyrosine hydroxylase; TNF: tumor necrosis factor; Trk: tropomyosin kinase

## Additional files

Additional file 1: Figure S1. The ROIs were determined manually, separating glandular epithelial tissue and stromal tissue (**a**). The nuclear morphometric and staining parameter enable the identification in epithelial cells (**b**). Gray levels of separated “blue staining channel” with segmented structures as an overlay (**c**). DAB staining mask as color labeled areas (**d**). Backward visualization positive stained cells (**e**), Backward visualization negative stained cells (**f**). (JPG 944 kb) Additional file 2: Figure S2. The selected endometriosis tissue required the presence of glandular epithelial cells and stromal cells. ROI were developed by selecting ‘custom’ mode applied to separate lumen, epithelial gland tissue and stromal tissue. The lumen ROI was excluded from analysis. *Black arrow* showing custom mode (**a**), ROI has been developed, *blue arrow* showing lumen, *red arrow* showing epithelial tissue, and *black arrow* showing stromal tissue. (JPG 282 kb) Additional file 3: Figure S3. Markers determination. Nucleus identification was determined by selecting blue color as marker. First, the markers button was chosen. Hematoxillin staining button was chosen. Color picker button was chosen. Blue color taken from the cell that may represent all nucleus. With the same procedure, brown color was chosen to detect brown color as result of DAB/IHC staining in cells. Blue color was chosen to detect hematoxillin staining in nucleus (**a**), brown color was chosen to detect result of IHC staining in cells (**b**). (JPG 203 kb) Additional file 4: Figure S4. Setting parameter for nuclei size. The nucleus size was determined as depicted in Fig. 4a. It was based on blue color detection (hematoxylin staining). Brown color expressed by cell was restricted from interior radius −4,81 μm to exterior radius 25.3 μm (**a**) and brown color resulted from IHC staining (**b**). (JPG 191 kb) Additional file 5: Figure S5. Scatter gram showing the result of hematoxylin staining (**a**) and DAB staining (**b**). The last step was determination of the immune positive or negative expression resulting from IHC staining. The ‘cut off’ option was used to set new values for one axis (y axis in DAB staining). The cut off menu was set at 18 and only for DAB staining and applied to all ROI (**b**). (JPG 150 kb) Additional file 6: Figure S6. Manual counting of nerve fibers. Region of interests were selected randomly and marked with a border to obtain an area of 1 mm^2^ each. A single nerve fiber was marked manually by using a tool available in Histoquest® software. (JPG 565 kb) Additional file 7: Figure S7 . Endometriosis lesion in submucosa of colon stained with antibody anti PGP9.5 (**a**), p75 (**b**), and TrkB (**c**) and anti TrkA (**d**). Red arrow shows nerve fiber, black arrow shows ganglion-like form. Original magnification × 200. Scale bare, 100 μm. (JPG 713 kb)
