# Supplementary figures and images for: Localization of TrkB and p75 receptors in peritoneal and deep infiltrating endometriosis: an immunohistochemical study
Source: Reprod Biol Endocrinol. 2016 Aug 12;14:43. doi: 10.1186/s12958-016-0178-5 (PMC4982126; doi:10.1186/s12958-016-0178-5)

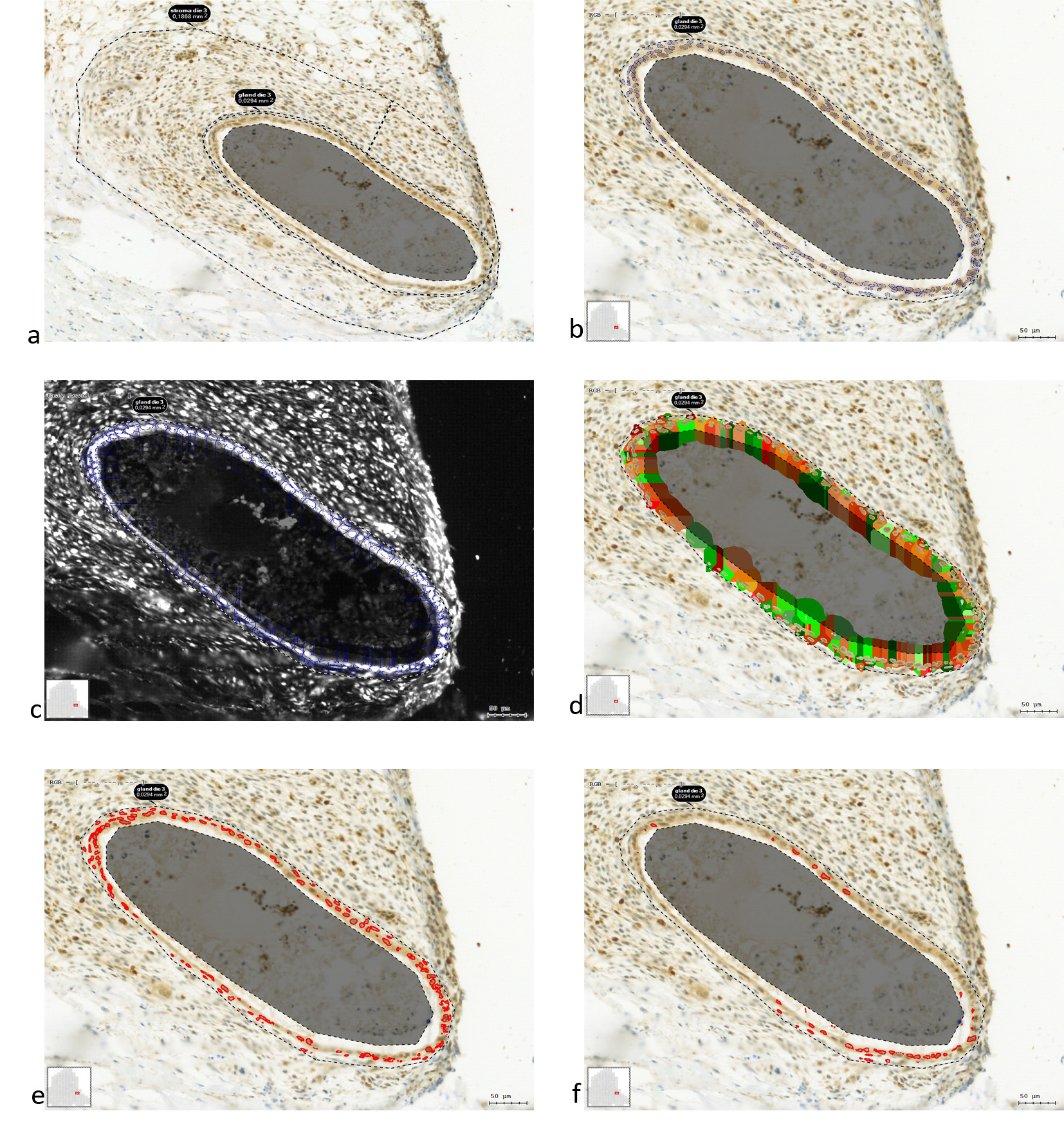

Supplement: Additional file 1: Figure S1. — The ROIs were determined manually, separating glandular epithelial tissue and stromal tissue (a). The nuclear morphometric and staining parameter enable the identification in epithelial cells (b). Gray levels of separated “blue staining channel” with segmented structures as an overlay (c). DAB staining mask as color labeled areas (d). Backward visualization positive stained cells (e), Backward visualization negative stained cells (f). (JPG 944 kb) [file 12958_2016_178_MOESM1_ESM.jpg]

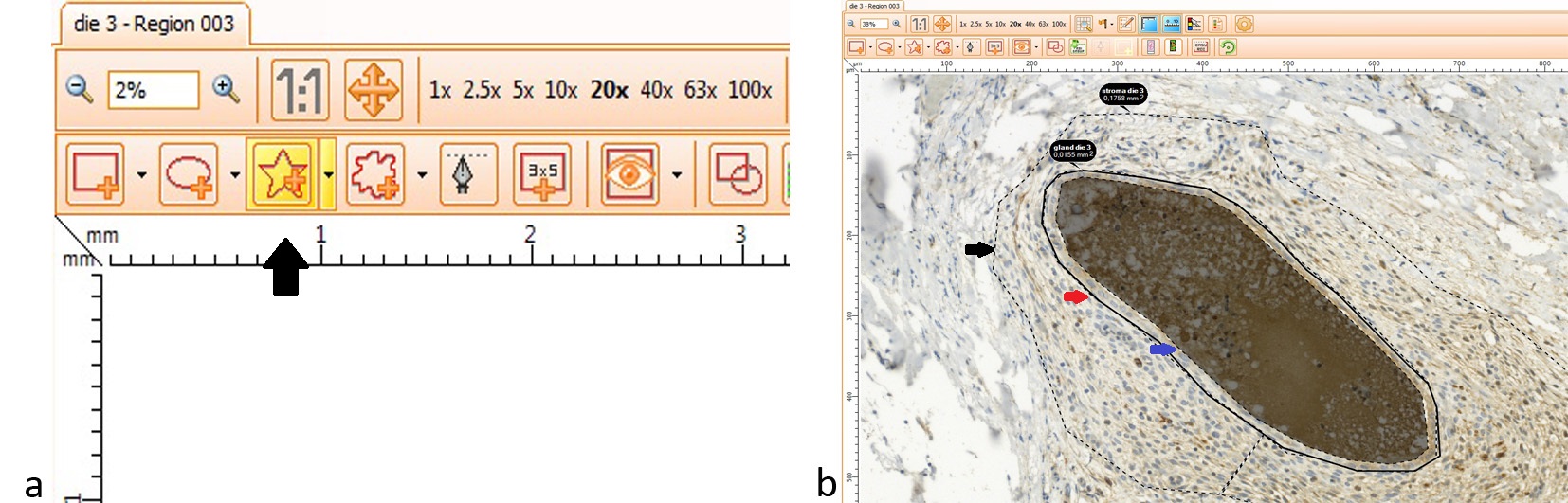

Supplement: Additional file 2: Figure S2. — The selected endometriosis tissue required the presence of glandular epithelial cells and stromal cells. ROI were developed by selecting ‘custom’ mode applied to separate lumen, epithelial gland tissue and stromal tissue. The lumen ROI was excluded from analysis. Black arrow showing custom mode (a), ROI has been developed, blue arrow showing lumen, red arrow showing epithelial tissue, and black arrow showing stromal tissue. (JPG 282 kb) [file 12958_2016_178_MOESM2_ESM.jpg]

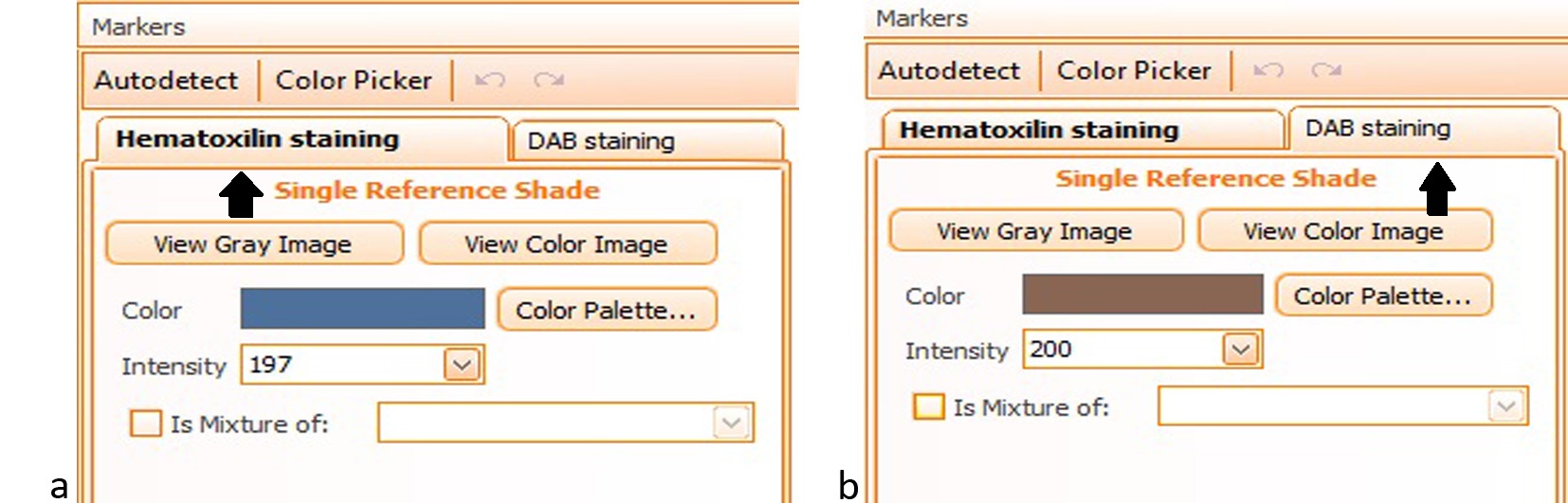

Supplement: Additional file 3: Figure S3. — Markers determination. Nucleus identification was determined by selecting blue color as marker. First, the markers button was chosen. Hematoxillin staining button was chosen. Color picker button was chosen. Blue color taken from the cell that may represent all nucleus. With the same procedure, brown color was chosen to detect brown color as result of DAB/IHC staining in cells. Blue color was chosen to detect hematoxillin staining in nucleus (a), brown color was chosen to detect result of IHC staining in cells (b). (JPG 203 kb) [file 12958_2016_178_MOESM3_ESM.jpg]

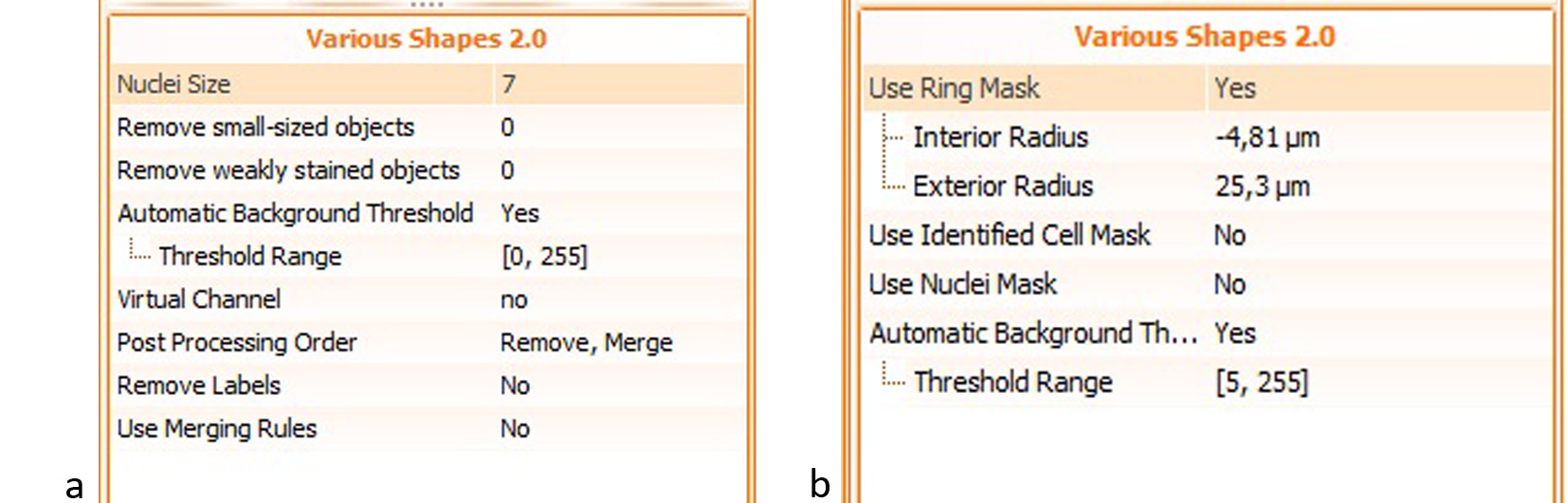

Supplement: Additional file 4: Figure S4. — Setting parameter for nuclei size. The nucleus size was determined as depicted in Fig. 4a. It was based on blue color detection (hematoxylin staining). Brown color expressed by cell was restricted from interior radius −4,81 μm to exterior radius 25.3 μm (a) and brown color resulted from IHC staining (b). (JPG 191 kb) [file 12958_2016_178_MOESM4_ESM.jpg]

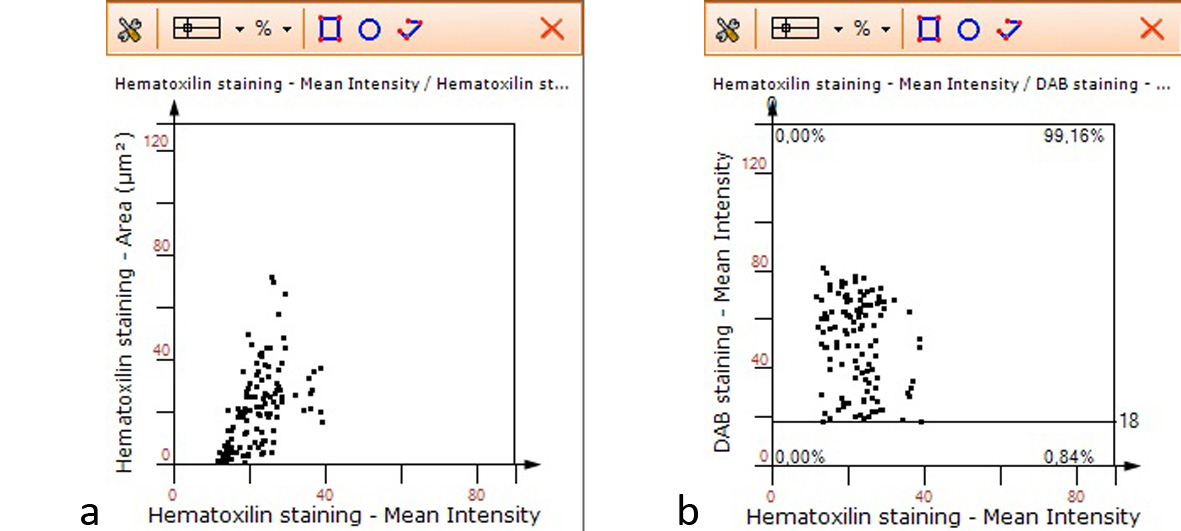

Supplement: Additional file 5: Figure S5. — Scatter gram showing the result of hematoxylin staining (a) and DAB staining (b). The last step was determination of the immune positive or negative expression resulting from IHC staining. The ‘cut off’ option was used to set new values for one axis (y axis in DAB staining). The cut off menu was set at 18 and only for DAB staining and applied to all ROI (b). (JPG 150 kb) [file 12958_2016_178_MOESM5_ESM.jpg]

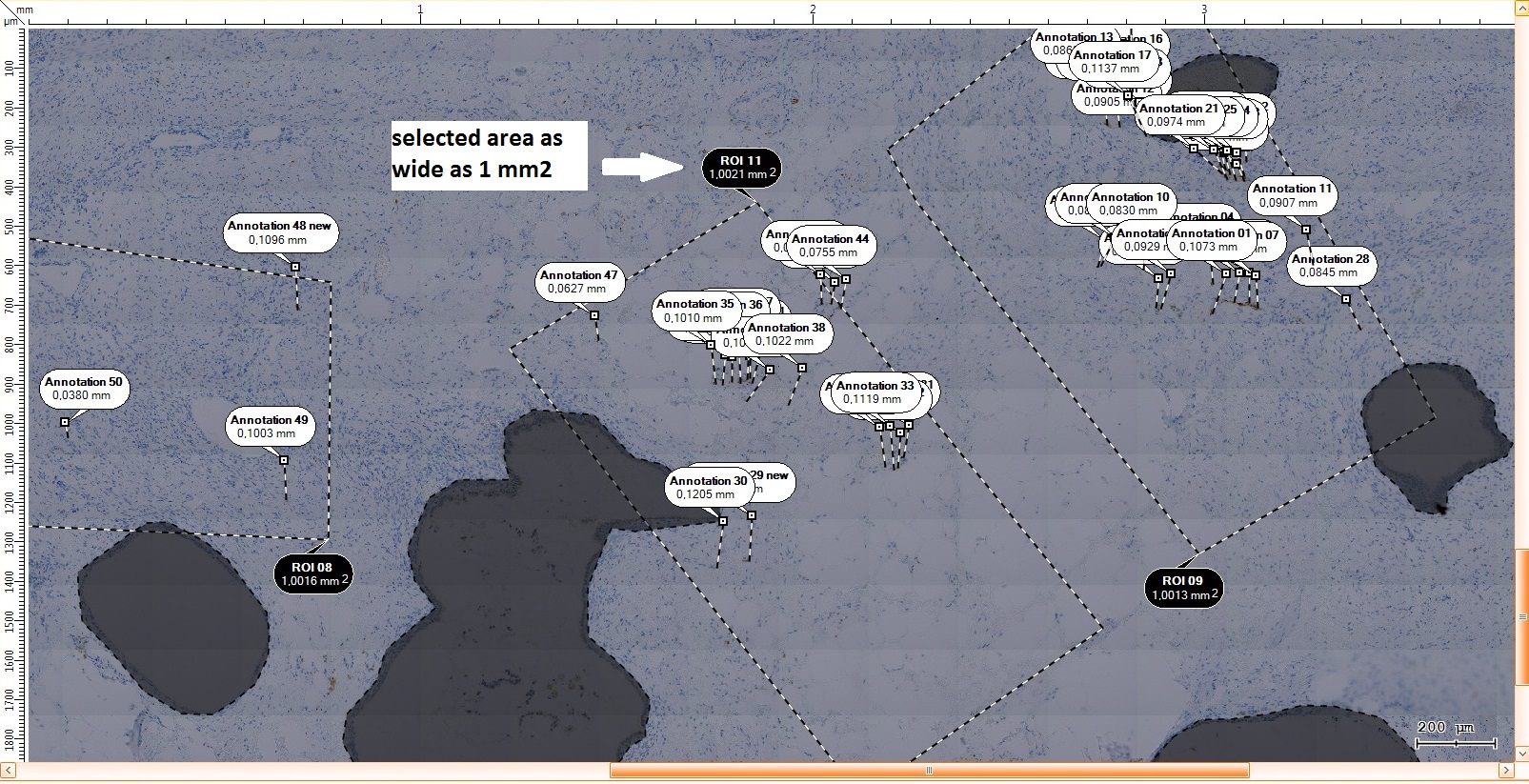

Supplement: Additional file 6: Figure S6. — Manual counting of nerve fibers. Region of interests were selected randomly and marked with a border to obtain an area of 1 mm2 each. A single nerve fiber was marked manually by using a tool available in Histoquest® software. (JPG 565 kb) [file 12958_2016_178_MOESM6_ESM.jpg]

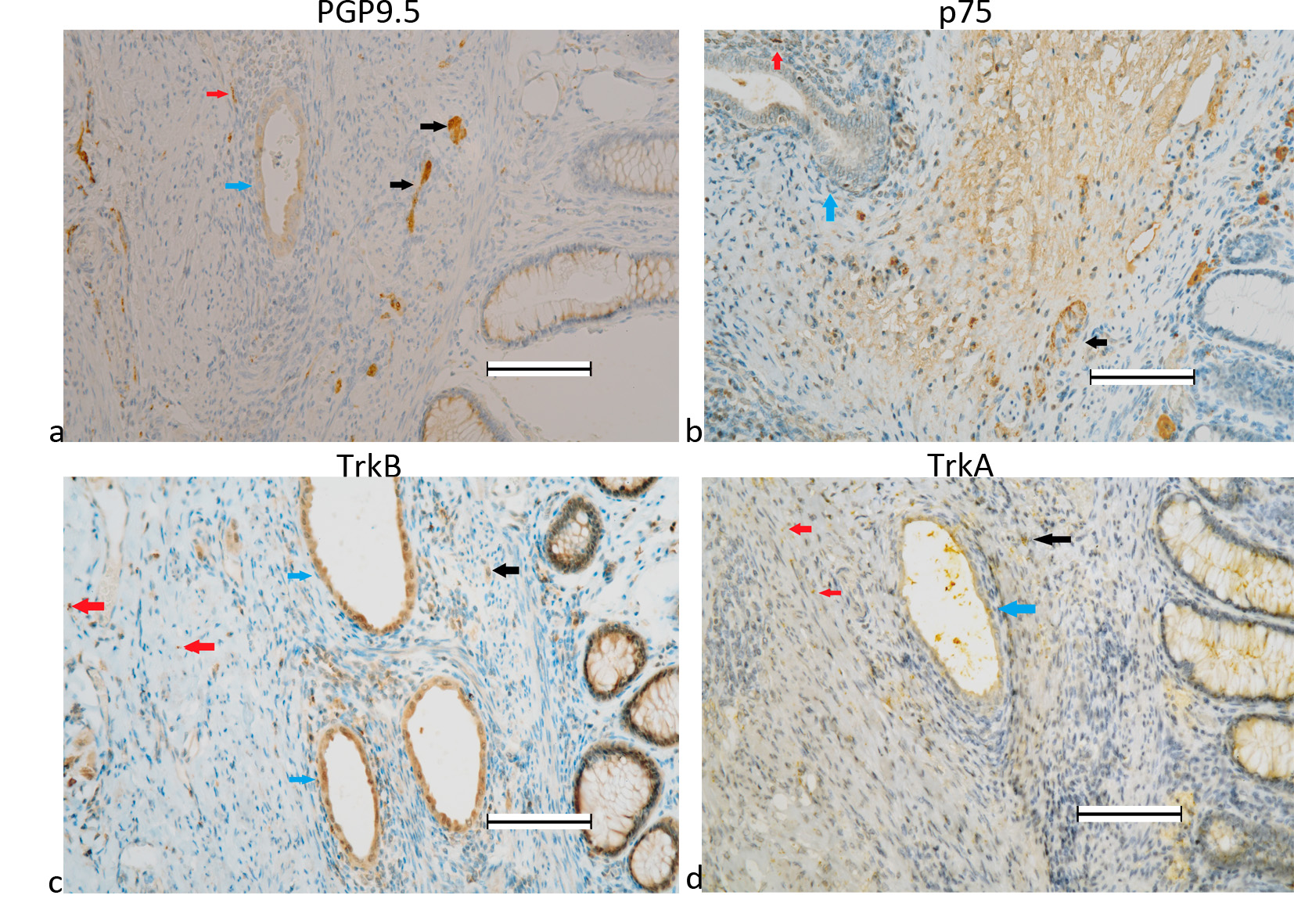

Supplement: Additional file 7: Figure S7 . — Endometriosis lesion in submucosa of colon stained with antibody anti PGP9.5 (a), p75 (b), and TrkB (c) and anti TrkA (d). Red arrow shows nerve fiber, black arrow shows ganglion-like form. Original magnification × 200. Scale bare, 100 μm. (JPG 713 kb) [file 12958_2016_178_MOESM7_ESM.jpg]
